# Class in the Medical College

**Published:** 1866-10

**Authors:** 


### Class in the Medical College.

From present indications, the next class in the University of Buffalo will be larger than ever before; the preliminary term, now nearly completed, gives promise of its being unusually large and intelligent. The increasing popularity of this school is sufficient evidence of the energy and capacity of its professorial staff, which, so far as we are informed, remains unchanged. The importance of ophthalmology, laryngoscopy, orthopædia and other divisions of medicine and surgery demand increased attention—at least some of these subjects could, no doubt, be more fully introduced with great advantage to the classes, even if by so doing, portions of the former curriculum of teaching was somewhat interfered with. These subjects are the attractive topics of professional observation and pursuit at the present time, and to pass them over almost unnoticed, is to forget the living present and study the classical and historical past. This institution is now wealthy in essential strength, and can afford to assume any elevation of standard it chooses. Its teachers understand better than any one else, what it' and other similar institutions most need.

